# Characterization of the GRAS gene family reveals their contribution to the high adaptability of wheat

**DOI:** 10.7717/peerj.10811

**Published:** 2021-02-23

**Authors:** Yanfeng Liu, Wei Wang

**Affiliations:** 1 School of Resources and Environmental Engineering, Ludong University, Yantai, Shandong, China

**Keywords:** Wheat, GRAS, Neofunctionalization, Subfunctionalization, Biotic and abiotic stress, Gene expression

## Abstract

GRAS transcription factors play important roles in many processes of plant development as well as abiotic and biotic stress responses. However, little is known about this gene family in bread wheat (*Triticum aestivum*), one of the most important crops worldwide. The completion of a quality draft genome allows genome-wide detection and evolutionary analysis of the GRAS gene family in wheat. In this study, 188 *TaGRAS* genes were detected and divided into 12 subfamilies based on phylogenetic analyses: DELLA, DLT, HAM, LISCL, SCL3, SCL4/7, SCR, SHR, PAT1, Os19, Os4 and LAS. Tandem and segmental duplications are the main contributors to the expansion of *TaGRAS*, which may contribute to the adaptation of wheat to various environmental conditions. A high rate of homoeolog retention during hexaploidization was detected, suggesting the nonredundancy and biological importance of *TaGRAS* homoeologs. Systematic analyses of *TaGRAS* indicated the conserved expression pattern and function of the same subfamily during evolution. In addition, we detected five genes belonging to the LISCL subfamily induced by both biotic and abiotic stresses and they may be potential targets for further research through gene editing. Using degradome and ChIP-seq data, we identified the targets of miR171 and histone modifications and further analyzed the contribution of epigenetic modification to the subfunctionalization of *TaGRAS*. This study laid a foundation for further functional elucidation of *TaGRAS* genes.

## Introduction

Bread wheat (*Triticum aestivum,* AABBDD) is a major food crop worldwide. Wheat is derived from two allopolyploidization events of three diploid progenitor species: *Triticum urartu* (AA), an *Aegilops speltoides*-related grass (SS≈BB) and *Aegilops tauschii* (DD) (Pont et al., 2019). Therefore, bread wheat has a huge and complex genome, making genomic studies challenging. However, the completion of a high-quality draft genome greatly promotes the genome-wide research, such as studies on homoeolog expression bias in various developmental stages by RNA-seq, functional genetic element detection by epigenomic maps and ancestry tracing of modern wheat by exome sequencing (IWGSC, 2018; Krzywinski et al., 2009; Li et al., 2019b; Pont et al., 2019).

GRAS is a transcription factor family that has been identified in many plant species (Huang et al., 2015; Liu & Widmer, 2014; Tian et al., 2004; Xu et al., 2016). In soybean (*Glycine max*), 117 GRAS genes were identified, and these *GmGRAS* genes might have been under strong purifying selective pressures during evolution (Wang et al., 2020). Sixty-two GRAS members were observed in barley (*Hordeum vulgare* L.), and 74.2% of them were intron-free (To et al., 2020). GRAS originated from a horizontal gene transfer event from soil bacteria, which was one of the key molecular signals allowing the ancestors of terrestrial plants to adapt to the terrestrial environment (Cheng et al., 2019). It was named based on the first detected members GAI (gibberellic acid insensitive), RGA (repressor of GA1-3 mutant) and SCR (scarecrow) (Laurenzio et al., 1996; Peng et al., 1997; Silverstone, Ciampaglio & Sun, 1998). Most of the GRAS proteins consist of 400 to 700 amino acids, and share a conserved C-terminal GRAS domain. The GRAS domain is composed of five motifs: LHRI (leucine heptad repeat I), LHRII (leucine heptad repeat II), VHIID, SAW, and PFYRE. VHIID exists in all of GRAS proteins, and combines with LHRI and LHRII to form the LHRI-VHIID-LHRII complex, which may be vital in interactions between proteins (Hirsch & Oldroyd, 2014; Itoh et al., 2002). The variable N terminus contains subfamily-specific molecular recognition features in the intrinsically disordered region, which act as bait during molecular recognition events (Sun et al., 2011).

The classification of the GRAS family is slightly different based on phylogenetic relationships. Initially, GRAS proteins were divided into eight subfamilies in *Arabidopsis*, including LS, LISCL, HAM, PAT1, SCL3, SHR, SCR and DELLA (Tian et al., 2004). However, 117 *GmGRAS* genes were divided into nine subfamilies in soybean (*Glycine max*), including DELLA, HAM, LAS, LISCL, PAT1, SCL3, SCL4/7, SCR and SHR (Wang et al., 2020). *Brachypodium distachyon* included another subfamilies: DLT (Niu et al., 2019). There are three other subfamilies in *populous trichocarpa*: Os19, Os4 and PT20 (Liu & Widmer, 2014). In *Citrus sinensis*, 50 GRAS genes were divided into 11 subfamilies, and subfamily CsGRAS34 was sweet orange-specific (Zhang et al., 2019). The most common expansion mechanisms of the GRAS gene family are tandem and segmental duplications, that might involve neofunctionalization, subfunctionalization or specialization during evolution, which play important roles in functional divergence (Huang et al., 2015; Keller et al., 2020; Liu et al., 2018).

GRAS proteins were found to play diverse roles in plant development. However, members of the same subfamily have conserved protein structures and may have similar biological functions. It is now well known that DELLA is a negative regulator of the GA (gibberellin) signaling pathway, and GA can release its inhibition by degrading DELLA (Hirano, Ueguchi-Tanaka & Matsuoka, 2008; Willige et al., 2007). In wheat, the development and utilization of the wheat semidwarfing allele *Rht1* (*reduced height1*, including *Rht-B1* and *Rht-D1*), which belongs to the DELLA subfamily, triggered a world-famous “Green Revolution” and spectacularly increased wheat yield. *Rht1* alleles were not only used to increase lodging resistance under high fertilizer applications, but also to enhance the harvest index by increasing grain numbers per ear (Tan, Koval & Ghalayini, 2013; Wu et al., 2011). Reduced height in wheat carrying *Rht-B1b* or *Rht-D1b* was the result of reduced sensitivity to GA resulting from base substitution (Peng et al., 1999). RGL2 participated in flower and ovule development through the GA pathway in *Arabidopsis* (Gómez et al., 2019). SCR and SHR positively regulate the formation of root radial organization. They participate in cortex/endodermal formation, and mutation of *SCR* or *SHR* leads to only a single cell layer between the epidermis and pericycle. *SCR* regulates cell division but not cortex and endodermis specification, while *SHR* participates in both processes (Haelariutta et al., 2000; Laurenzio et al., 1996). In *Salvia miltiorrhiza*, the SHR member *SmGRAS1* is preferentially expressed in the root periderm, and activates *SmKSL1* expression by directly binding to the GARE motif in its promoter , thus promoting tanshinone biosynthesis (Li et al., 2019a). *BrLAS* is primarily expressed in roots and axillary meristems. Overexpression of the *Brassica rapa* gene *BrLAS* in Arabidopsis plants resulted in delayed bolting, flowering time, senescence, decreased fertility and enhanced drought resistance (Li et al., 2018b).

In addition, GRAS participates in light signal transduction. Expression level reduction of *TaSCL14*, a member of the LISCL subfamily, leads to dwarfed plants, decreased photosynthetic capacity, earlier leaf senescence and reduced resistance to photooxidative stress (Chen et al., 2015). Phytochrome photoreceptors enable plants to sense divergent signals to adapt to various environmental changes. The GRAS family proteins PAT1 and SCL13 act as mediators in the light signaling pathway. SCL13 is a positive regulator in response to continuous red light in the downstream of phyB (phytochrome B). In *Arabidopsis*, downregulation of *SCL13* displayed reduced sensitivity to red light (Torres-Galea et al., 2006). *PAT1* is involved in the signal transduction of phyA, and null-function mutation of *PAT1* leads to reduced resposes to continuous far-red light (Bolle, Koncz & Chua, 2000). In addition, downregulation of the PAT1 subfamily gene, *GRAS2*, decreases fruit weight by restraining cell expansion in tomato (*Solanum lycopersicum*) (Li et al., 2018a). Morever, GRAS controls tillering in rice. For example, mutation of GRAS family gene *MOC1* (*Monoculm1*) results in the failure of tiller buds formation, and plants have only a main culm (Li et al., 2003). Two GRAS proteins, NSP1 (nodulation signaling pathway 1) and NSP2, promote the initiation of nodules, and miR171h targets NSP2 to prevents the overcolonization of arbuscular mycorrhizal fungi in roots (Lauressergues et al., 2012; Murakami et al., 2006). miR171 cleavaed the transcripts of *HAM1*, and disruption of this process induces abnormal embryogenesis, leading to the abnormal phenotypes in seedling shoots including fused cotyledons, asymmetric tricots or no cotyledons (Takanashi et al., 2018).

Although great progress has been made in the identification of GRAS proteins in plants, to the best of our knowledge, the evolution and expression profiles of the entire GRAS family in wheat have not been described in detail. In this study, we detected *TaGRAS* genes genome-wide and performed a comprehensive analysis of their phylogeny, gene structure, chromosomal location and expression patterns in different developmental stages and under various stresses. In addition, we analyzed epigenetic regulation of *TaGRAS* expression. This work provides valuable information for further functional studies in wheat.

## Materials & Methods

### Genome-wide detection and sequence analysis of *TaGRAS*

The CDS and protein sequences of both high and low confidence bread wheat (*Triticum aestivum* cv. Chinese Spring) genes were downloaded from https://urgi.versailles.inra.fr/download/iwgsc/IWGSC_RefSeq_Annotations/v1.0/. High-confidence genes indicated genes with complete gene models (genes contain both start and stop codons) and very good sequence homology to plant proteins from SwissPort and Poaceae proteins in trEMBL but no good sequcence homology in transposon database TREP. Low-confidence genes indicated that genes which have incomplete gene models but high identity to plant proteins from SwissProt; or genes with complete gene models but no significant homology to TREP, UniPoa or Unimagdatabases; or incomplete gene models with very high identity to the annotated Poaceae protein in SwissProt or trEMBL but not in the TREP database. Besides, low confidence genes including genes with incomplete gene models and no significant homology to any genes in SwissProt, trEMBL or TREP database. The complete and detailed parameters can be referred to the previous literature (IWGSC, 2018). The GRAS protein sequences of *Arabidopsis* (32), rice (*Oryza sativa,* 56), barley (*Hordeum vulgare* L., 62) and *Brachypodium distachyon* (48) were retrieved from previous studies (Niu et al., 2019; Tian et al., 2004; To et al., 2020). These sequences were merged together to query bread wheat proteins by BLASTp. Proteins with Evalue ≤ 1e−5, identity ≥60 and length coverage ≥50% were used to detect the exaistance of GRAS domains with HMMER 3.0 and the Hidden Markov Model (HMM) profile of the GRAS domain (PF03514, http://pfam.xfam.org/, Evalue ≤1e−5) (Finn et al., 2016; Mistry et al., 2013). Then, these candidate *GRAS* genes were examined to confirm the existence of GRAS domain in SMART (http://smart.embl-heidelberg.de/), PFAM (http://pfam.xfam.org/) and Conserved Domain Database (CDD) in NCBI (https://www.ncbi.nlm.nih.gov/). Proteins with truncated, lacking, or anonymous GRAS domain were eliminated. The confirmed *GRAS* genes were named based on an abbreviation for the species name *Triticum aestivum* (*Ta*) and their order on the chromosomes. The subcellular localization of GRAS proteins were predicted in WoLFPSORT (https://wolfpsort.hgc.jp/). Exon-intron structures were constracted in Gene Structure Display Server (GSDS, http://gsds.cbi.pku.edu.cn). ExPASy (https://web.expasy.org/compute_pi/) was used to calculate isoelectric point and molecular weight. Phylogenetic tree was constructed with MEGA 5.0 based on the maximum likelihood method using 1000 bootstraps (Hall, 2013). The phylogenetic tree was subsequently visualized in iTOL (https://itol.embl.de/). Homoeologous genes between the A, B and D subgenome of wheat were detected based on the phylogenetic tree and previous classifications (Ramírez-González et al., 2018; Schilling et al., 2020). The chromosomal location was displayed with circos (Krzywinski et al., 2009). Ka (non-synonymous substitution) and Ks (synonymous substitution) was calculated using ParaAT (Parallel Alignment and back-Translation) software with MA methods (Zhang et al., 2012).

### Expression profiles in different tissues and under stress conditions

The expression level (TPM, transcripts per million) of *TaGRAS* genes were downloaded from http://www.wheat-expression.com/, and the heatmap was generated with R packages “*pheatmap*” (scale=“none”, cluster_rows=F, cluster_cols=F). *TaGRAS* genes were classified as 10 clusters with kmeans according to expression level, and then the clusters were divided into groups that preferentially expressed in reproductive, vegetative or ubiquitous tissues. Homoeolog expression patterns were performed as previously described (Krzywinski et al., 2009). Briefly, TPM of triad with 1:1:1 ratio between the three subgenomes was extracted, and the triads with summed TPM lower than 0.5 were discarded. The euclidean distance of normalized expression level of triads was calculated and the triads were classified based on the shortest distance as *A*∕*B*∕*D* dominant, *A*∕*B*∕*D* suppressed or balanced profiles. Ternary plot was generated with R package “*vcd*”.

### ChIP-seq data analysis

The ChIP-seq raw data (H3K27ac, H3K27me3, H3K36me3, H3K4me1, H3K4me3, H3K9ac and H3K9me2) were downloaded from Gene Expression Omnibus Database (accession: GSE121903) (Li et al., 2019b). Trimmomatic was used to remove the reads with low quality. Then the clean reads were mapped to the reference sequence of bread wheat (IWGSC, v1.0) with bowtie2 (Bolger, Lohse & Usadel, 2014; IWGSC, 2018). To ensure the accuracy of results, reads from PCR duplication or with mapping quality below 20 were removed. The reads-enriched regions (peaks) of each mark were detected with MACS (v1.3.7) (Zhang et al., 2008). The peaks with at least 1 base pair overlapped between biological replicates were identified as the credible peaks. Target genes were defined as genes with a peak in the gene body. The TPM data of corresponding stage seedling were downloaded from http://www.wheat-expression.com/, (Table S7). Genes with TPM higher than 0.5 were recorded as expressed genes.

### Degradome data analysis

To detected the targets of miR171, the degradome data of 21 and 28 DPA (days post anthesis) grains were downloaded from NCBI Gene Expression Omnibus (accession: GSE65799) (Zhang et al., 2015a). The degradome data of seed embryos, seedling leaves, seedling roots and grains of 8 days after pollination were downloaded from NCBI SRA database (accession: SRP040143) (Sun et al., 2014). First of all, low-quality reads were removed with Trimmomatic (Bolger, Lohse & Usadel, 2014). CleaveLand4 was used to predictthe targets of miR171, the targets with alignment score above 4.5 were removed (Addo-Quaye, Miller & Axtell, 2009).

## Results

### Detection of *TaGRAS* in bread wheat

In this study, 188 *TaGRAS* genes were detected and their molecular weights varied from 26.69 to 164.81 kDa (Table S1). These genes were named from *TaGRAS1* to *TaGRAS188* according to their chromosomal location. The longest TaGRAS protein was TaGRAS82, which had 819 amino acids, while the shortest was TaGRAS172 with only 245 amino acids. The subcellular localization of TaGRAS proteins were predicted by WoLFPSORT. 42.0% (79/188) of TaGRAS proteins were predicted to be located in nucleus. As previously confirmed, TaGRAS179 (TaMOC1-7A) was predicted to be located in the cell nucleus (Zhang et al., 2015b). The isoelectric point of TaGRAS proteins ranged from 4.73 to 10.21, of which 158 were below 7.00, suggesting that most TaGRAS were slightly acidic, and different TaGRAS proteins played roles in different microenvironment.

### Phylogenetic and gene structure analysis of *TaGRAS* gene families

**Figure 1:**
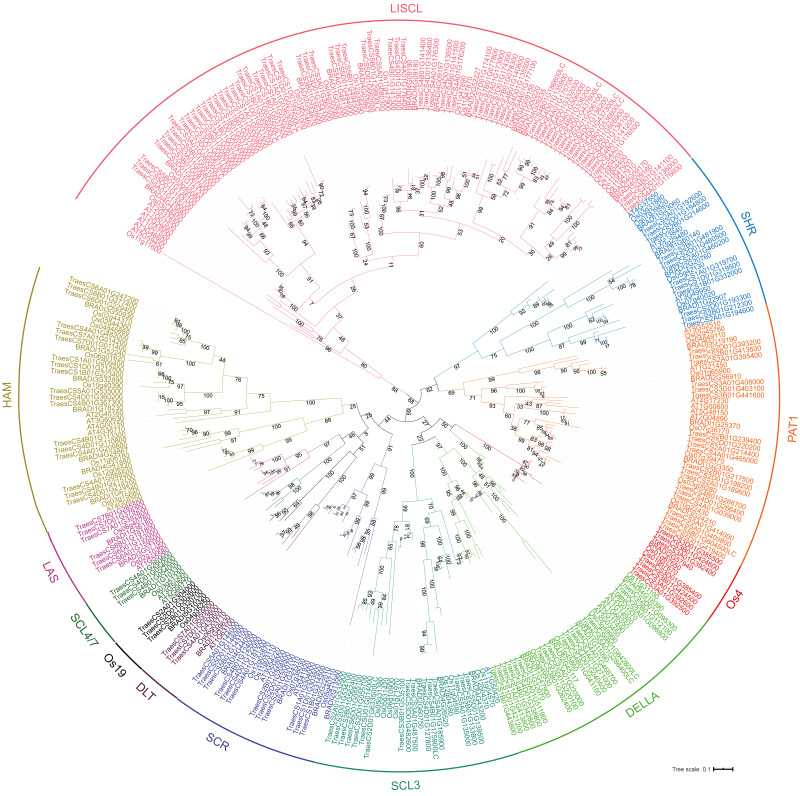
Phylogenic tree of GRAS proteins from bread wheat, Arabidopsis, rice and *Brachypodium distachyon*. The protein sequences of GRAS from bread wheat, Arabidopsis, rice and *Brachypodium distachyon* were used to perform multiple sequence alignment with ClustalW and MEGA 5.0 was used to generate phylogenic tree. The subfamilies were indicated with different colors.

**Figure 2:**
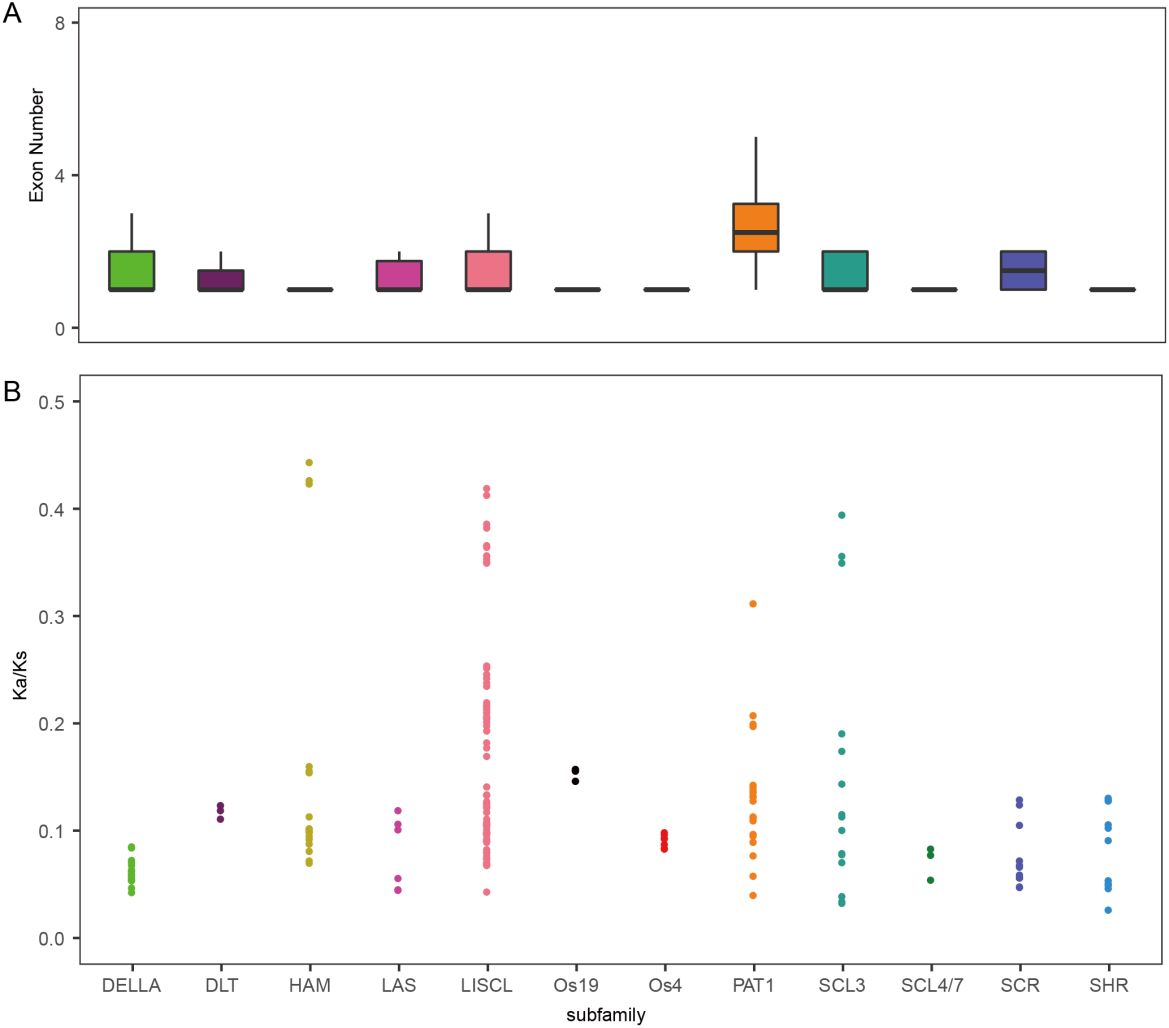
Exon number and Ka/Ks analysis of TaGRAS. ** (**A) Exon number of *TaGRAS* subfamilies (*, *P* < 0.05, Wilcoxon rank sum test). (B) Distribution of Ka/Ks in subfamilies of TaGRAS.

To analyze the evolutionary relationship of the GRAS protein in *Arabidopsis*, *B. distachyon*, rice and wheat, a phylogenetic tree was constructed using the maximum likelihood method with MEGA5.0 (Hall, 2013) (Fig. 1). *TaGRAS* genes were divided into 12 subfamilies: SHR (containing 12 members), PAT1 (20), DELLA (18), LISCL (70), SCL3 (16), DLT (3), SCR (12), LAS (6), SCL4/7 (3), HAM (18), Os4 (7) and Os19 (3). Consistent with the exon-intron structure in grapevine, rice and tomato, 60.0% (114/188) of *TaGRAS* genes had no intron (Fig. S1) (Huang et al., 2015). The exception was the subfamily PAT1, which had a significantly higher number of exons than other subfamilies (Fig. 2A). When considering the Ka/Ks, none of the *TaGRAS* genes were under positive selection. The genes from subfamily DELLA, LAS, SCL4/7, SCR and SHR were under strict purifying selection pressure compared with PAT1and LISCL (Fig. 2B).

### Chromosomal location of *TaGRAS*

**Figure 3:**
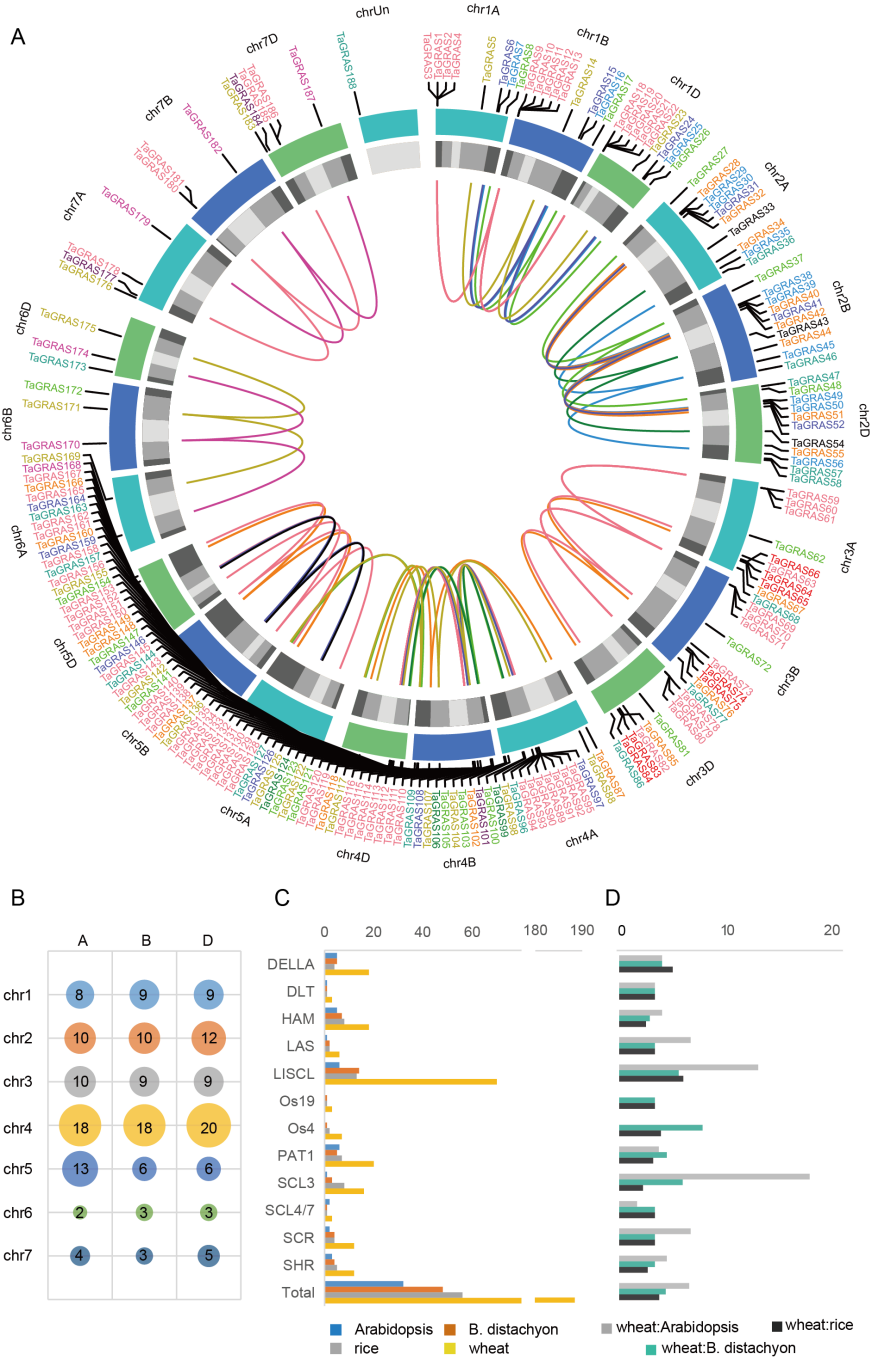
Chromosomal location and number of TaGRAS subfamily genes. (A) The chromosomal location of *TaGRAS*. The subfamily genes were indicated with same color as Fig. 1. The outer track indicated each chromosome, and the inner track indicated chromosomal segment (Light grey: C; grey: R2a and R2b; dark grey: R1 and R3). The inner links indicated homoeologous genes. (B) TaGRAS gene number located on each chromosome. (C) Gene number of each subfamily in wheat, rice, Arabidopsis and *Brachypodium distachyon*. (D) Gene number ratio of each subfamily is shown for wheat: rice, wheat: Arabidopsis and wheat: *Brachypodium distachyon*.

A total of 188 *TaGRAS* genes unevenly distributed on wheat chromosomes with homoeologous group 4 had a higher density of genes, which was significantly higher than expected (56/188, 29.8%; *χ*^2^test, *P* < 0.001; Figs. 3A, 3B). The enrichment in homoeologous group 4 was mainly the result of tandem duplication (two *TaGRAS* genes were separated by no more than three genes, Table S1). For example, *TaGRAS89-94*, *TaGRAS111-116* and *TaGRAS129-135* were tandemly duplicated clusters on chromosomes 4A, 4B and 4D respectively. In addition, the presence of *TaGRAS* in chromosomal segment C was significantly less frequent than all wheat genes (data from Ramírez-González et al., 2018), as only two subfamilies had genes located in this region, indicating that they were preferentially distributed on the distal end of chromosomes (1.60% vs 10.67%; *χ*^2^test, *P* < 0.001; Table S2). In contrast, in subfamily LAS, no gene was located in distal telomeric segments (segment R1 and R3, Table S2), indicating a subfamily-specific location pattern.

### *TaGRAS* genes exhibit a high rate of homoeolog retention

When considering the total number of *GRAS* genes, the wheat *GRAS* gene number was higher than that of *B. distachyon*, rice or Arabidopsis even when polyploid level was considered (188/3>48, 56, 32, Fig. 3C). The increase was mainly the result of amplification of the LISCL, DELLA and PAT1 subfamilies. Gene duplication events contributed to the expansion of these subfamilies. For example, the BRADI1G15123 gene from the LISCL subfamily of *B. distachyon* was sister to 23 wheat genes. This was the result of segmental and tandem duplication in the ancestor species of *Triticum* (before polyploidization of wheat). In some cases, the expansion was supported by low-confidence genes, such as BRADI3G24210, which was sister to four wheat genes, including one low-confidence gene (*TaGRAS160*, TraesCS5B01G604500LC, Fig. 1).

To further explore the evolution of *TaGRAS* genes during the polyploidization of wheat, we detected the homoeologous relationship in detail. As shown by the phylogenetic tree, in 71.4% (35/49) of the subclades, one *B. distachyon* gene and one rice gene were closely related to a triad of wheat homoeologs (genes had a 1:1:1 correspondence across the three A, B and D subgenomes) (Fig. 1). For example, Os03g15680 and BRADI1G67340 were sister to the triads *TaGRAS88* (TraesCS4A01G088500), *TaGRAS117* (TraesCS4B01G215700) and *TaGRAS136* (TraesCS4D01G216200). We noted that *TaGRAS* genes of subfamilies SCL4/7, LAS, DLT and SCR all presented as triads. Moreover, compared with all of annotated wheat genes (data from Ramírez-González et al., 2018), *TaGRAS* had a higher proportion of genes belonging to triads (79.8% vs 35.8%) (Table S3), suggesting a high rate of homoeolog retention during hexaploidization.

### Gene expression profiles of *TaGRAS* in various tissues

**Figure 4:**
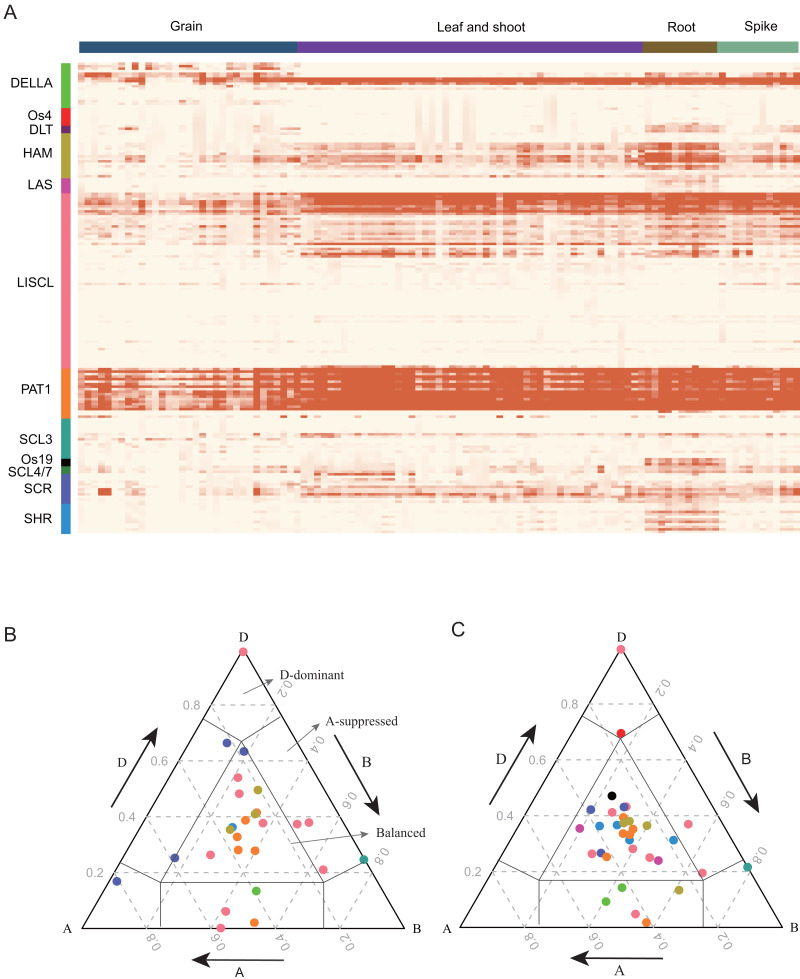
Expression pattern (TPM) of *TaGRAS*. (A) Gene expression profile of *TaGRAS* in different developmental stage. Heatmap was generated with R package “pheatmap” using parameter scale=“none”. The TPM datas was shown in Table S4. (B) Ternary plot showing relative expression abundance of *TaGRAS* for triads with 1:1:1 ratio in leaves. Each circle represents a gene triad, and its A, B and D coordinates consist of the relative contribution of each homologous gene to the expression of the overall triad. Triads in vertices means single-subgenomic-dominant class, for example the D-dominant region, while triads near edges and between vertices indicated suppressed classes, for example the A-suppressed region. Triads in the center region indicated balanced category. The color of each subfamily was same with color bar in (A). (C) Ternary plot showing relative expression abundance of *TaGRAS* for triads with 1:1:1 ratio in roots.

GRAS proteins are transcriptional regulators that play important roles in plant growth and development (Cheng, 2004; Fleck & Harberd, 2002; Hirsch & Oldroyd, 2014; Tyler et al., 2004). To detect the expression pattern of *TaGRAS*, RNA-seq data were analyzed. Approximately 69.7% of *TaGRAS* were expressed in at least one developmental stage (131/188, TPM>0.5, Fig. 4A, Table S4). The majority of genes from the same subfamily displayed similar expression patterns. In general, the gene expression levels of subfamily PAT1, except for *TaGRAS149,* were much higher than those of other subfamilies (Fig. 4A, Table S4). In addition, genes from subfamily PAT1 were expressed at high lever in all of the investigated tissues, although there was a slight decrease in grains (Fig. 4A, Table S4). Os19, DLT and SHR subfamily genes were preferentially expressed in roots, consistent with their roles in root development. All of the genes from LAS were hardly expressed in different tissues except for the quite low level in roots (Fig. 4A, Table S4). We further hierarchically clustered the genes according to TPM as previously described (Schilling et al., 2020). Fig. S2 showed that the largest subfamily LISCL contained genes from nine clusters, which were preferentially expressed in ubiquitous, reproductive, vegetative tissues or even not expressed. Subfamily DLT preferentially expressed in vegetative tissues (Fig. S2).

As 79.8% of *TaGRAS* presented as triads, we explored the homoeolog expression bias in leaf/shoot, root, spikelet and grain. We mainly focused on genes with summed expression levels above 0.5 TPM across the traid. According to the expression level and the euclidean distance of A, B and D subgenome homoeologs, the expression bias was classified as balanced, A/B/D-surpressed or A/B/D-dominant patterns. Consistent with all of wheat genes (data from Ramírez-González et al., 2018), 62.4% of the triads of roots showed similar relative abundances from the A, B and D subgenome, namely, the balanced category (Table S5) (Ramírez-González et al., 2018). In reproductive tissue spikelets and grains, approximately 60.7% of the triads were assigned to the balanced category (Figs. 4B, 4C, Fig. S2 and Table S5). In addition, in contrast to previous reports, a higher proportion of D-homoeolog suppression was observed across all the tissues (16.2% vs 5.7%). That mainly results from three triads: PAT1 (*TaGRAS148-TaGRAS160-TaGRAS166*), DELLA (*TaGRAS100-TaGRAS105-TaGRAS123*) and LISCL (*TaGRAS150-TaGRAS161-TaGRAS167*), which displayed D-homoeolog suppression in all detected tissues (Table S5).

### *TaGRAS* genes play roles under biotic and abiotic stresses

**Figure 5:**
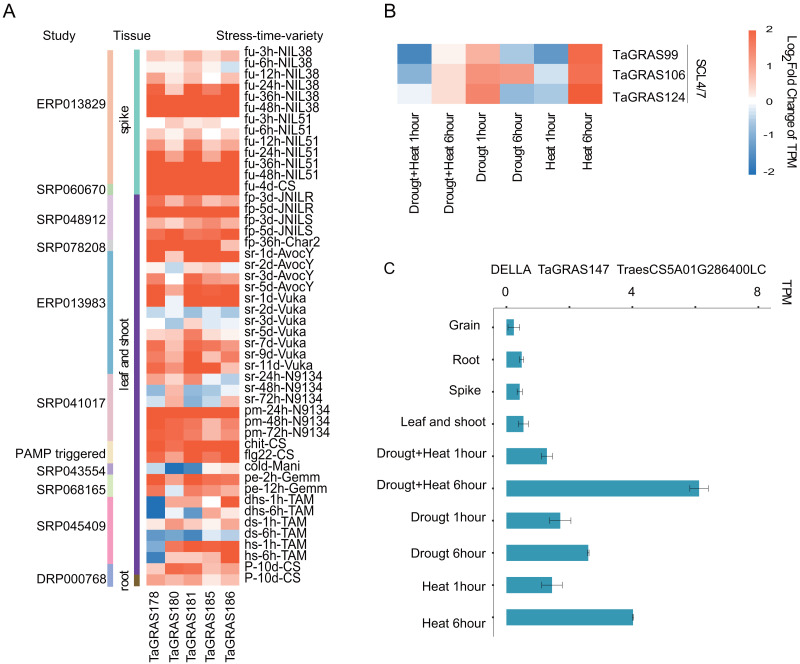
Expression pattern of *TaGRAS* under abiotic and biotic stresses. (A) Expression level change of five genes from LISCL subfamily under stresses. The original data accession number and the tissues were shown. fu: fusarium graminearum inoculation; fp: fusarium pseudograminearum inoculation; sr: stripe rust pathogen; pm: powdery mildew pathogen; chit: chitin (1 g per liter) treatment; flg22: flg22 (500 nM) treatment; cold: cold 2 weeks (4); pe: PEG 6000; dhs: drought and heat stress; ds: drought stress; hs: heat stress; P: phosphorous starvation 10 days. (B) Expression level change of SCL4/7 subfamily genes under heat or/and drought stresses. (C) Expression level of *TaGRAS147* in different tissue and abiotic stresses. The TPM datas were shown in Table S5.

To explore the function of *TaGRAS* genes in response to stresses, the TPM data of bread wheat genes under various treatments were downloaded and analyzed (Table S6). We noted that five LISCL homoeolog genes, *TaGRAS178*, *TaGRAS180*, *TaGRAS181*, *TaGRAS185* and *TaGRAS186*, were induced by various stress treatments (Fig. S3). They were gradually induced after inoculation with *Fusarium graminearum* (accession number, ERP013829, Fig. 5A). In addition, they showed significant upregulation in leaves and shoots when infected with stripe rust pathogens, powdery mildew pathogens, chit or flg22 (Fig. 5A). Moreover, abiotic stress, such as phosphorous starvation, heat and drought stress or PEG6000 treatment, induced their expression (Fig. 5A). These results imply that these genes may play an important role in various diseases and abiotic resistance. Three genes of subfamily SCL4/7 were mainly expressed in roots and upregulation was detected in leaves after 6 h of heat stress treatment (Fig. 5B). A low-confidence gene from subfamily DELLA, *TaGRAS147*, was expressed at a very low level in the developmental stages, but showed upregulation after 6 h of heat, drought and both stresses, suggesting that the authenticity of the gene (Fig. 5C).

### Epigenetic modification regulated the expression of *TaGRAS*

**Figure 6:**
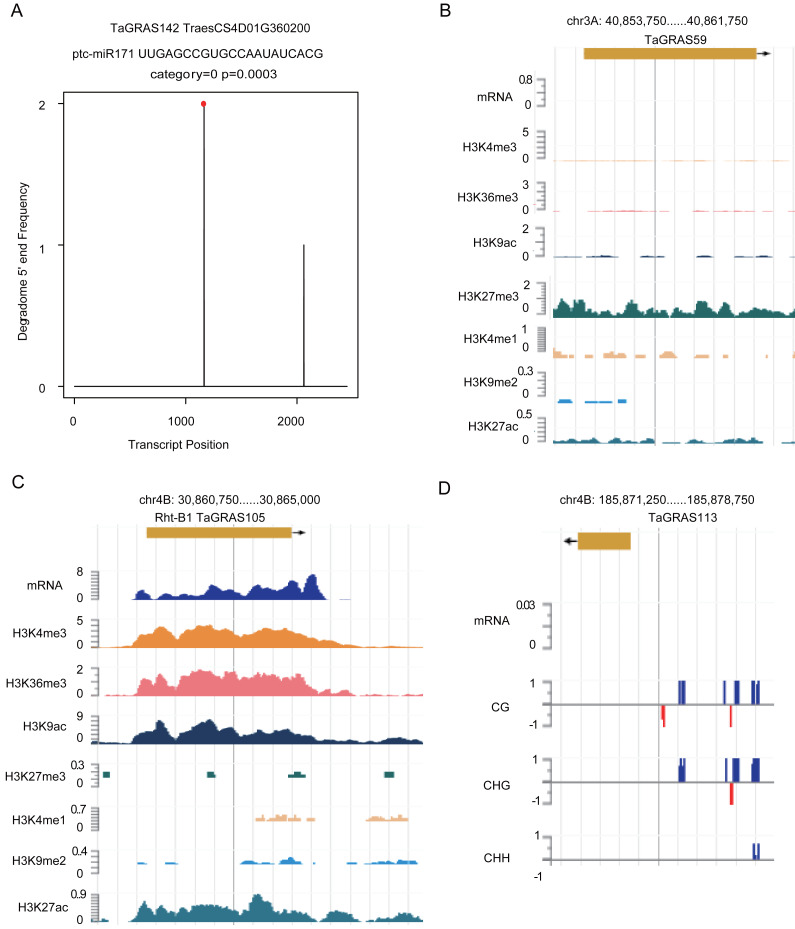
Epigenetic modifications regulate expression level of *TaGRAS* genes. (A) T-plot shows the target of miR171. (B–C) Histone modifications targeted *TaGRAS*. (D) DNA methylation modification level in the upstream of *TaGRAS113*.

According to a previous study, *GRAS* genes are the targets of miR171 (Llave et al., 2002). Using degradome data of grains, *TaGRAS142*, *TaGRAS155*, *TaGRAS169* and *TaGRAS171* were predicted to be targets of miR171 (Fig. 6A, Fig. S4 and Table S4). In leaves, miR171 was found to target *TaGRAS104*, *TaGRAS175* and *TaGRAS183* (Table S4). In addition to miRNAs, other epigenetic modifications play important roles in regulating the expression of genes. Here, we analyzed genes regulated by histone modifications in leaves. Consistent with the function of H3K9me2 in inhibiting the activation of transposons in centromere regions, none of the *TaGRAS* genes were modified by H3K9me2 (Table S7) (Ayyappan et al., 2015; Kowar et al., 2016). As a repressive mark, H3K27me3 modified 25.0% (32/128) of the nonexpressed *TaGRAS* (TPM<0.5), such as *TaGRAS37* (Fig. 6B, Table S7) (Molitor et al., 2014). In contrast, approximately half of the expressed *TaGRAS* were targets of H3K36me3, H3K4me3 and H3K9ac (48.3%, 50.0% and 50.0%, respectively, Table S7). This result was comparable with their enrichment at transcript start sites of activated genes (Du et al., 2013; Liu et al., 2010; Yang, Howard & Dean, 2014). For example, *Rht-B1* was modified by H3K4me3, H3K36me3, H3K9ac and H3K27ac in leaves (Fig. 6C, Table S7). Several nonexpressed genes were modified by neither miRNA nor histone modifications. These genes may be regulated by DNA methylation (Fig. 6D). Certainly, genetic factors are also crucial for gene expression regulation, such as cis regulatory elements and mutations, which require further study.

## Discussion

In this study, we detected 188 *TaGRAS* genes and divided them into 12 subfamilies. As previously reported, most of the *TaGRAS* had no introns except for the genes from the PAT1 subfamily. *TaGRAS* was unevenly distributed on chromosomes, with a specifically high density of homoeologous group 4.

According to the RNA-seq data, many wheat *TaGRAS* genes have evolutionarily conserved functions. First, the expression patterns of *TaGRAS* genes were comparable with their homologs in rice and Arabidopsis or other plants. For example, *SHR* and *SCR* in Arabidopsis were reported to regulate cortex/endodermal specification (Haelariutta et al., 2000; Laurenzio et al., 1996). These subfamily genes of wheat exhibited similar pattern as they preferentially expressed in root, suggesting that they may play conserved roles in regulating radial pattern formation (Fig. 4). *DLT* negatively regulates the grain size of rice by regulating the number of cells in glume and affecting the development of palea and lemma (Sun et al., 2013). *DLT* is expressed in wheat spikelets as well. *RHT1* in wheat is a homolog of rice *SLR* and maize *D8*, both of which are negative modulators in the GA signaling pathway, and utilization of these genes in breeding significantly increased the yield (Asano et al., 2009; Lawit et al., 2010). Second, *TaGRAS* exhibited a conserved pattern between homoeologs in wheat. *TaGRAS* displayed a high rate of homoeolog retention and a balanced expression pattern in the triads. A total of 62.4% of *TaGRAS* genes belonged to the balanced category in roots, and it is noteworthy that all of the SCR and SHR subfamily genes were equally expressed in the three subgenomes, suggesting their nonredundant role in root development (Table S5). The conserved expression pattern and high rate of homoeolog retention imply the significance of the *TaGRAS* family in plant development.

The GRAS subfamily genes participate in regulating a wide range of pathways. The function of DELLA in GA signaling in Arabidopsis and rice has been well described (Wang & Deng, 2013). It is vital for the trade-off between adaptation to disadvantageous environments and plant growth. In general, the accumulation of DELLA reduced the growth of plants and concentrated the resources of plants on stress resistance, while the degradation of DELLA mediated by GA promoted the growth under favorable conditions (Van De Velde et al., 2017). PAT1 is specific for the phyA signaling pathway in Arabidopsis, and *PeSCL7* improved drought and salt resistance in *Populus euphratica* (Bolle, Koncz & Chua, 2000; Ma et al., 2010). Members of the HAM subfamily control shoot meristem maintenance in *Petunica hybrid* (Stuurman, Jaggi & Kuhlemeier, 2002). In wheat, tandem and segmental duplication events lead to expansion of the *TaGRAS* family, especially the subfamilies PAT1, DELLA and LISCL. The expansion of these subfamilies may be helpful for improving the adaptability of wheat to various environmental conditions due to their vital roles in plant development. In wheat, most of the PAT1 subfamily genes were expressed at a relatively high level in different developmental stage, and five LISCL subfamily genes were induced by both biotic and abiotic stress (Fig. 4 and Table S6). These genes may be potential targets for gene editing in further research.

Bias in homoeolog expression potentially represents an early trend of sub- or neofunctionalization of duplicated gene copies (Ramírez-González et al., 2018). D-homoeolog suppression in the triads *TaGRAS148-TaGRAS160-TaGRAS166* (PAT1) and *TaGRAS150-TaGRAS161-TaGRAS167* (LISCL) seemed to result from the neofunctionalization of D-homoeolog. Although hardly expressed in all the detected tissues, *TaGRAS166* was induced by phosphorus starvation (accession number, DRP000768) and infection with stripe rust pathogen or powdery mildew pathogen (accession number, SRP041017) in leaves, and *TaGRAS167* was induced by *Fusarium pseudograminearum* inoculation in spikes (accession number, SRP060670), while their homoeologs were relatively stable under stress. Neofunctionalization may be one of the mechanisms of adaption to environmental changes.

In *Arabidopsis*, miR171 cleaves mRNAs of *SCL6*, *SCL22* and *SCL27*, and miR171-GRAS modules are critical for shoot apical meristem and root indeterminacy maintenance (Llave et al., 2002; Schulze et al., 2010). Overexpression of miR171c or mutation of its targets leads to reduced shoot branching, shorter primary roots, accumulation of chlorophyll and abnormal leaf and flower formation (Wang et al., 2010). *SlGRAS24*, a member of the HAM subfamily in tomato, is the target of miR171 (Huang et al., 2015). Overexpression of *SlGRAS24* resulted in shorter and narrower leaves, abnormal axillary bud emergence, a reduced fruit-set ratio and delayed flowering (Huang et al., 2017). Here, we detected seven subfamily HAM genes as targets of miR171 by degradome data. Moreover, we detected the targets of several histone modifications. Activating marks, such as H3K4me3, H3K9ac and H3K27ac, regulated the highly expressed genes, while the repressive mark H3K27me3 modified genes that were not expressed or expressed at a very low level (Table S7). Epigenetic modifications may play a role in the subfunctionalization of *TaGRAS* genes. The conclusion is reflected in the triad of the SCR subfamily: *TaGRAS31*, *TaGRAS41* and *TaGRAS52*. The protein identity between these genes was higher than 98%, suggesting the limited genetic diversity. On the other hand, we observed that in seedling leaves, H3K36me3 modified *TaGRAS31* and *TaGRAS52* but not *TaGRAS41* (Table S7). Meanwhile, *TaGRAS 4 1* was hardly expressed in the leaves, leading to the B-homoeolog suppression of this triad (Table S5). The limited genetic variation and difference in H3K36me3 modification levels indicate that epigenetic regulation contributed to the subfunctionalization of *TaGRAS41* in leaves.

In conclusion, *TaGRAS* genes are vital for wheat development and have significant potential value in wheat molecular breeding improvement. Our research provides a theoretical basis for further functional research on *TaGRAS* genes.

## Conclusion

Hexaploidy and abundant repetitive sequences result in bread wheat having a large and complex genome, which makes the whole-genome analysis challenging. *GRAS* genes are important for wheat development. Based on our data, we speculate that *GRAS* gene duplications might have been crucial in increasing the adaptability of wheat to different environmental conditions. By transcriptome analysis, we provide a basis for the identification of gene-editing targets to improve wheat performance. Furthermore, epigenetic modifications participated in the neo- and subfunctionalization of *TaGRAS* genes, which is another direction for evolutionary analysis.

##  Supplemental Information

10.7717/peerj.10811/supp-1 Supplemental Information 1 Phylogenetic relationships and gene structures of theTaGRAS genes of wheat

10.7717/peerj.10811/supp-2 Supplemental Information 2 Expression cluster analysis of TaGRAS genes.

10.7717/peerj.10811/supp-3 Supplemental Information 3 Expression level change (Log_2_(stress+0.5)/(control+0.5)) of all the TaGRAS genes under biotic and abiotic stresses

10.7717/peerj.10811/supp-4 Supplemental Information 4 T-plot shows the target of miR171 in seeds (upper) and seedling leaves (bottom)

10.7717/peerj.10811/supp-5 Supplemental Information 5 List of all *TaGRAS* genes identified in bread wheat

10.7717/peerj.10811/supp-6 Supplemental Information 6 Location of TaGRAS genes on respect chromosome segment

10.7717/peerj.10811/supp-7 Supplemental Information 7 Homoeologous group of *TaGRAS* genes

10.7717/peerj.10811/supp-8 Supplemental Information 8 TPM datas of different developmental stage and tissues for Fig. 4A

10.7717/peerj.10811/supp-9 Supplemental Information 9 Homoeolog expression bias for triads in leaf/shoot, root, spekelet and grain

10.7717/peerj.10811/supp-10 Supplemental Information 10 TPM of *TaGRAS* in control and various stress treatment

10.7717/peerj.10811/supp-11 Supplemental Information 11 Regulation of *TaGRAS* by histone modifications

10.7717/peerj.10811/supp-12 Supplemental Information 12 The CDS sequences of *TaGRAS*

## Additional Information and Declarations

### Data Availability

The following information was supplied regarding data availability: Raw data, including the gene ID, homoeologous group, gene expression level, the targets of histone modifications, and the CDS sequences of TaGRAS, are available in the Supplemental Files.
